# Amphiregulin Regulates Phagocytosis-Induced Cell Death in Monocytes via EGFR and Matrix Metalloproteinases

**DOI:** 10.1155/2018/4310419

**Published:** 2018-11-04

**Authors:** Christopher Platen, Stephan Dreschers, Lucy Kathleen Reiss, Jessica Wappler, Thorsten W. Orlikowsky

**Affiliations:** ^1^Department of Neonatology, University Children's Hospital, Aachen, Germany; ^2^Institute of Pharmacology and Toxicology, Medical Faculty, RWTH Aachen University, Aachen, Germany; ^3^Molecular Tumor Biology, Department of General, Visceral and Transplantation Surgery, University Hospital, Aachen, Germany

## Abstract

Neonates are highly susceptible to microbial infections which is partially attributable to fundamental phenotypic and functional differences between effector cells of the adult and neonatal immune system. The resolution of the inflammation is essential to return to tissue homeostasis, but given that various neonatal diseases, such as periventricular leukomalacia, necrotizing enterocolitis, or bronchopulmonary dysplasia, are characterized by sustained inflammation, newborns seem predisposed to a dysregulation of the inflammatory response. Targeted apoptosis of effector cells is generally known to control the length and extent of the inflammation, and previous studies have demonstrated that phagocytosis-induced cell death (PICD), a special type of apoptosis in phagocytic immune cells, is less frequently triggered in neonatal monocytes than in adult monocytes. We concluded that a rescue of monocyte PICD could be a potential therapeutic approach to target sustained inflammation in neonates. The EGFR ligand amphiregulin (AREG) is shed in response to bacterial infection and was shown to mediate cellular apoptosis resistance. We hypothesized that AREG might contribute to the reduced PICD of neonatal monocytes by affecting apoptosis signaling. In this study, we have examined a cascade of signaling events involved in extrinsic apoptosis by using a well-established *in vitro E. coli* infection model in monocytes from human peripheral blood (PBMO) and cord blood (CBMO). We found that CBMO shows remarkably higher pro-AREG surface expression as well as soluble AREG levels in response to infection as compared to PBMO. AREG increases intracellular MMP-2 and MMP-9 levels and induces cleavage of membrane-bound FasL through engagement with the EGF receptor. Our results demonstrate that loss of AREG rescues PICD in CBMO to the level comparable to adult monocytes. These findings identify AREG as a potential target for the prevention of prolonged inflammation in neonates.

## 1. Introduction

Infectious diseases affect people of all ages, but newborns are an exceptionally susceptible group. Every year, an estimated 4 million neonates die in the first month of life, while about one third of deaths are caused by infection [1]. This increased susceptibility of newborns to infection is mainly due to an inappropriate and biased immune response [2] which is in part attributable to phenotypic and functional differences between the immune effector cells of neonates and adults. Monocytes play a central role in host defence by eliminating pathogens via phagocytosis and orchestrating the subsequent immune reaction [3]. However, their insufficient elimination from the inflammatory site could destroy the balance between amplification and resolution of inflammation. Indeed, monocytosis has been shown to be associated with widespread inflammatory conditions [4] and a prolonged monocyte response contributes to the pathogenesis of inflammatory diseases [5]. One mechanism that may contribute to an effective termination of inflammation by controlling the length and extent of the monocyte reaction is a special form of effector cell apoptosis referred to as phagocytosis-induced cell death (PICD). Strikingly, evidence suggests that PICD is less frequently triggered in neonatal monocytes as compared to adult monocytes although both exhibit identical phagocytic and intracellular degradation activities [6]. This finding is of central importance since various studies presented a correlation between the prolongation of inflammation and typical diseases in neonates such as periventricular leukomalacia [7], necrotizing enterocolitis [8], or bronchopulmonary dysplasia [9]. These detrimental sequelae can furthermore contribute to the development of diseases in adult age including rheumatoid arthritis, multiple sclerosis, and Parkinson's disease [10] which highlights the need for assessing the mechanisms of inflammation in the neonatal period. We concluded that a rescue of neonatal monocyte PICD might be a potential therapeutic approach to reduce inflammation in newborns and target typical neonatal inflammatory diseases.

Type II cytokine amphiregulin (AREG) was first described as a member of the epidermal growth factor family almost 30 years ago [11]. Originally specified as epithelial cell-associated factor, AREG is nowadays known to be expressed in a variety of activated immune cells [12] including monocytes [13]. AREG is synthesized as a transmembrane precursor referred to as pro-AREG, while sequential proteolytic cleavage of the precursor results in the release of the soluble AREG protein [14]. Cleavage of pro-AREG is essentially mediated by the tumor-necrosis factor-alpha converting enzyme (TACE) also known as ADAM-17, which is a member of the disintegrin and metalloproteinase (ADAM) family [15]. AREG triggers signaling through engagement with the EGF receptor (EGFR), leading to a cascade of signaling events essential for a variety of fundamental cellular processes including metabolism, cell cycle, and proliferation [16]. Intriguingly, AREG was shown to contribute to many of the “hallmarks of cancer” and was extensively described to mediate apoptosis resistance in several epithelial cancer cells [17, 18]. Beside its function in tumorigenesis, AREG has been demonstrated to be involved in the pathogenesis of inflammatory processes such as rheumatoid arthritis and chronic airway diseases [19]. As previous studies demonstrated host cell transcriptional upregulation of AREG in response to bacterial infections with *Shigella flexneri*, enterohemorrhagic *Escherichia coli*, and *Helicobacter pylori* [20–22] as well as increased proteolytic cleavage of pro-AREG upon infection with *Neisseria gonorrhoeae* [23], we hypothesized that AREG might contribute to the reduced PICD of neonatal monocytes by affecting extrinsic apoptosis signaling.

The extrinsic apoptosis signaling cascade depends on transmembrane receptor-mediated interactions of several members of the tumor necrosis factor (TNF) receptor gene superfamily [24]. One well-characterized ligand and corresponding receptor of this superfamily is FasL/FasR (CD95L/CD95) [25]. Binding of the ligand to FasR leads to the recruitment of cytoplasmic adapter proteins which bind to the receptor via corresponding death domains. The formation of this death-inducing signaling complex mediates the autocatalytic activation of procaspase-8 [26], resulting in the execution phase of apoptosis [27]. The induction of apoptosis by FasL/FasR interaction is particularly triggered if FasL is bound to the plasma membrane and not soluble [28, 29]. A link between FasL and AREG was already demonstrated by a study in hepatocytes which proves AREG to reduce cellular apoptosis via shedding of membrane-bound FasL [30]. The conversion into the soluble form is mediated by cleavage of the membrane-bound ligand via the matrix metalloproteinases MMP-9 [31] and MMP-2 [32]. The expression of MMP-9 and MMP-2 is regulated by the mitogen-activated protein kinase (MAPK) signaling pathway [33, 34], which is in turn regulated by EGFR [35].

To better understand the underlying mechanisms involved in PICD and to investigate whether the difference in PICD could be caused by a differentially regulated AREG/EGFR signaling, we used a well-established *in vitro* infection model. Primary monocytes from peripheral blood (PBMC) and cord blood (CBMC) were isolated from human donors and were infected *in vitro* with the model organism *E. coli* which has been identified as one of the most common causes of mortality of neonates [36]. A cascade of events involved in extrinsic apoptosis signaling was analyzed which unveiled evidence for a functional involvement of AREG in the reduced PICD of neonatal monocytes and suggested AREG as potential target for the modulation of neonatal inflammation.

## 2. Material and Methods

### 2.1. Patients

Written consent was obtained from adult participants for using their venous blood. All neonates were delivered vaginally and did not show signs of infection, ensured by controlling white blood cell count, interleukin-6, C-reactive protein, and the clinical status. Excluded from the study were mothers with (1) amnion infection or (2) prolonged labour (>12 hours) as well as (3) preterm infants before 36 weeks of gestation and (4) small for gestational age neonates. After cord ligation, umbilical cord blood was immediately placed in heparin-coated tubes (10 IU/ml blood). All experimental procedures were approved by the Ethics Committees of Aachen University Hospital (Permission No. EK150/09, October 6, 2009).

### 2.2. Mononuclear Cell Cultures

Isolation of peripheral blood mononuclear cells (PBMC) and cord blood mononuclear cells (CBMC) was performed by using Ficoll density gradient centrifugation (Amersham, Freiburg, Germany). Cells were washed and resuspended in VLE-RPMI 1640 (Biochrom, Berlin, Germany). To separate the monocytes, magnetic cell sorting monocyte isolation kit II (Miltenyi Biotec, Bergisch Gladbach, Germany) was used according to the manufacturer's protocol. The minimal purity of the resulting population was defined to be 90% CD14-positive cells, as detected by flow cytometry. For analysis of postphagocytic reactions, 10^6^ cells/ml were seeded in 24-well cell culture plates (Costar, Bodenheim, Germany) in VLE-RPMI 1640 medium supplemented with 10% heat-inactivated fetal bovine serum (FBS, Biochrom, Germany) and 1% penicillin/streptomycin (PenStrep, Thermo Fischer, Massachusetts, USA).

### 2.3. Microorganisms

*E. coli* (strain BL21) transformed with the vector pGEX-4T-1, which contains the recombinant red fluorescent protein (dsRed) gene, were a kind gift from Prof. L. Rink (Institute of Immunology, RWTH Aachen University, Germany) [37]. *E. coli* DH5*α*, an encapsulated K12 laboratory strain, as well as *E. coli* dsRed were grown in Lennox-L-Broth-medium (Thermo Fisher, Massachusetts, USA) until early logarithmic phase and were used immediately. *S. agalactiae* was a kind gift from Prof. M. Hornef (Institute of Medical Microbiology, RWTH Aachen University, Germany). After growing overnight on blood agar plates, *Streptococcus agalactiae* (*S. agalactiae*, group B streptococcus) was resuspended in PBS. Bacterial infection of mammalian cells was performed at a multiplicity of infection (MOI) of 20 : 1 for *E. coli* and 25 : 1 for *S. agalactiae*. Culture medium without antibiotics was used during the infection process. *Candida albicans (C. albicans)* SC5314 were kindly provided by Prof. Joachim Morschhäuser (University of Würzburg, Germany). The yeast was grown overnight on 4% Sabouraud Dextrose Agar (Merck Millipore, Darmstadt, Germany) at 28°C and was afterwards diluted in 0.9% NaCl solution to a final concentration of 1 × 10^7^ cells/ml. Yeast infection of mammalian cells was performed at a MOI of 5 : 1.

The infection process was performed as follows: 1 × 10^6^ PBMC/CBMC/ml was incubated for 1 h with the bacteria/yeast and was then washed with FBS to remove remaining extracellular microorganisms. Afterwards, the infected cells were cultivated for 23 h to ensure examination of postphagocytic reactions 24 h after infection.

### 2.4. AREG Stimulation and Inhibitor Treatment

Recombinant human amphiregulin was purchased from R&D Systems (Minneapolis, USA), aliquoted in sterile PBS containing 0.1% BSA, and used in final concentrations of 0.5 or 0.05 *μ*g/ml. Neutralizing AREG antibody (clone AF262, R&D Systems, Minneapolis, USA) was used in a concentration of 1 *μ*g/ml. Chlorhexidine (CHX) was purchased from Santa Cruz (Dallas, USA) and aliquoted in DMSO used in a final concentration of 20 *μ*g/ml (0.002%). Control cells were vehicle-treated with the appropriate dose of DMSO. Neutralizing EGFR antibody (clone D1D4J, Cell Signaling, Frankfurt, Germany) was used in a dilution of 1 : 100. Treatment of cells was started 1 h prior to infection and was continued during the time of infection and the experiment.

### 2.5. Immunocytological Staining

Chamber slides (Millicell EZ Slides, Merck Millipore, Darmstadt, Germany) were coated with 10 *μ*g/ml poly-L-lysine (molecular weight 70,000–150,000, Sigma-Aldrich, Darmstadt, Germany) in PBS for 10 min at RT. Monocytes were isolated and afterwards infected with *E. coli* dsRed. PBMO/CBMO were seeded in a density of 5 × 10^6^ cells/cm^2^ to the slide and were incubated for 23 h. Cells were fixed with methanol for 15 min at −20°C and were afterwards blocked with 1% BSA and 0.3% Triton X-100 in PBS for 1 h at RT. AREG antibody was incubated overnight at 4°C at a dilution of 1 : 500, and the secondary antibody was incubated for 1 h at RT. Texas Red-X phalloidin (Thermo Fisher, Massachusetts, USA) was applied at a concentration of 1 : 1000 and was incubated 30 min at RT to stain F-actin. Zeiss Axioplan 2 with HBO 100 illuminator (Carl Zeiss, Oberkochen, Germany) with CoolSNAP HQ2 CCD Camera (Visitron Systems, Puchheim, Germany) as well as VisiView Imaging Software (Visitron Systems, Puchheim, Germany) was used to take fluorescence pictures. DyLight 488 was analyzed using filter set Zeiss FS60 (shift-free, excitation: 510/15, emission: 480/20, and magnification: 100-fold), and the Texas Red signal was analyzed using filter set Zeiss FS15 (shift-free, excitation: 546/12, emission: 590, and magnification: 100-fold). An overlay of pictures was manually performed using ImageJ software (US National Institutes of Health, Bethesda, USA).

### 2.6. Apoptosis Analysis

DNA fragmentation was performed according to Nicoletti et al. [38]. Separated monocytes were fixed with 2% paraformaldehyde in PBS for 2 h at RT and afterwards permeabilized by incubation in PBS-T (0.1% Triton X-100 in PBS) for 20 min at RT. Cells were resuspended in PBS-PI (70 *μ*g/ml propidium iodide (Carl Roth, Karlsruhe, Germany) with 13 units RNAse (New England Biolabs, Ipswich, USA) in PBS), incubated for 10 min at RT, and then analyzed by flow cytometry. Discrimination of cell doublets was implemented by assessing PI-width/PI-area as described [39]. Quantification of apoptotic cells was accomplished by plotting the number of events against the PI area, while apoptotic nuclei were ascertainable as hypodiploid DNA peak (sub-G1) as presented in Supplementary Figure 3.

### 2.7. Determination of Surface Antigens by Flow Cytometry

Samples were analyzed on a FACSCanto flow cytometer (Becton Dickinson, Mountain View, NJ, USA), which was calibrated daily. Cells were incubated with 10% FBS for 10 min on ice to prevent nonspecific binding. Afterwards, the cells were incubated with the antibodies or corresponding isotype controls in the concentration recommended by the manufacturer for 20 min on ice without light exposure. After washing the cells with PBS supplemented with 0.1% BSA, the samples were analyzed. Monocytes were gated using CD14 expression, forward scatter (FSC), and sideward scatter (SSC), and the data were analyzed applying FCS Express V4.0 research Edition software (De Novo Software, Glendale, California, USA).

### 2.8. ELISA

AREG and FasL concentrations in supernatants from monocyte cultures were determined by enzyme-linked immunosorbent assay (ELISA). Absorbance was measured in SpectraMax i3 Multi-Mode Microplate Reader (Molecular Devices, San Jose, USA) at 450 nm. For detection of AREG, the Human Amphiregulin ELISA Kit (Sigma-Aldrich, Darmstadt, Germany) was used according to the manufacturer's protocol. Measurement was executed with a range from 4 to 500 pg/ml and a sensitivity of <2 pg/ml. For detection of FasL, Human FasL PicoKine ELISA Kit (Boster Biological Technology, Pleasanton, USA) was used according to the manufacturer's instructions. Measurement was executed with a range of 15.6 pg/ml–1000 pg/ml and a sensitivity of <2 pg/ml.

### 2.9. Antibodies

All stainings were performed according to the manufacturer's recommended protocol. APC-labeled CD14 antibody (clone MEM-15) was purchased from ImmunoTools (Friesoythe, Germany). FITC-labeled FasL antibody (clone SB93a) was purchased from SouthernBiotech (Birmingham, USA), and FITC-labeled antibody to human MMP-9 (clone 56129) was purchased from R&D Systems (Minneapolis, USA); the corresponding mouse IgG2b kappa isotype control (clone eBMG2b) was obtained from eBioscience (Waltham, USA). Antibody against human EGFR IgG (clone D1D4J) was purchased from Cell Signaling (Frankfurt, Germany), and the corresponding FITC-labeled anti-rabbit IgG secondary antibody was obtained from BD Pharmingen (San Jose, USA). Antibody against human AREG IgG (clone AF262) was purchased from R&D Systems (Minneapolis, USA), and the corresponding secondary antibody DyLight 488 Anti-Goat IgG was obtained from Rockland (Limerick, USA). Antibody binding to human MMP-2 IgG1 (clone 101) was obtained from Invitrogen (Carlsbad, USA), and the corresponding FITC-labeled secondary antibody anti-mouse IgG1 (clone 1F8) was purchased from ImmunoTools (Friesoythe, Germany).

### 2.10. Statistical Analysis

GraphPad Prism 7 statistical software (GraphPad Software, La Jolla, USA) was used for analyses. Results are expressed as mean + standard deviation (SD). Data were analyzed using either one-way ANOVA or two-way ANOVA with Bonferroni's multiple comparisons test. Values of *p* < 0.05 were considered as statistically significant.

## 3. Results

### 3.1. Infection with *E. coli* Promotes Pro-AREG Shedding in Monocytes

**Figure 1:**
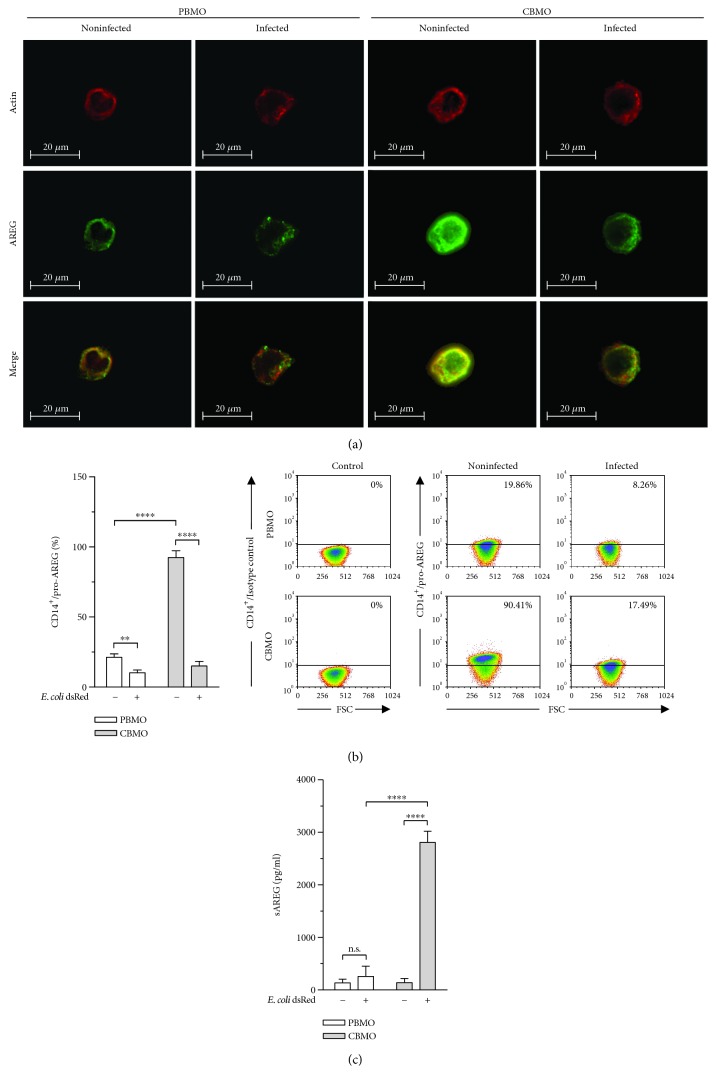
Neonatal monocytes display elevated pro-AREG surface expression and increased release of AREG in response to *E. coli* infection. PBMO and CBMO were incubated with *E. coli* for 1 h, extracellular bacteria were removed, and cells were cultivated for 24 h in total. (a) Immunocytological staining shows AREG protein expression (green) in monocytes. Texas Red-X phalloidin was applied to visualize F-actin in the cytoskeleton (red). (b) Pro-AREG surface expression in uninfected and *E. coli*-infected monocytes was quantified by using flow cytometry (*n* = 5). Representative dot plots show gating strategy and cutoff value for AREG expression. (c) sAREG levels in the supernatant of monocytes were quantified by using ELISA (*n* = 5). Data are shown as means + SD. Statistical analysis was performed using one-way ANOVA with Bonferroni's multiple comparisons test (ns: not significant, ^∗^ *p* < 0.05, ^∗∗^ *p* < 0.01, and ^∗∗∗∗^ *p* < 0.001).

While it was previously reported that AREG is expressed in monocytes of adult donors [13], the situation in neonates remains unknown. We were intrigued to note that CBMO showed a remarkably higher pro-AREG surface expression as compared to PBMO, while *E. coli* infection caused a clear reduction of pro-AREG in CBMO (Figure 1(a)). The immunocytological staining unveiled a noticeable difference between monocytes of adult and neonatal origin which was reproducible by quantitative flow cytometric analysis (Figure 1(b)). Since AREG could be internalized instead of being shed, we next quantified soluble(s) AREG concentrations in the supernatant of PBMO and CBMO by using ELISA (Figure 1(c)). Whereas sAREG levels remained almost unchanged in PBMO, *E. coli* infection significantly increased sAREG levels in CBMO, indicating plasma membrane shedding of pro-AREG. Strikingly, quantification revealed 11-fold higher sAREG levels in the supernatant of CBMO as compared to PBMO.

### 3.2. Infection with *E. coli* Induces Cell Surface Expression of EGFR in Monocytes

**Figure 2:**
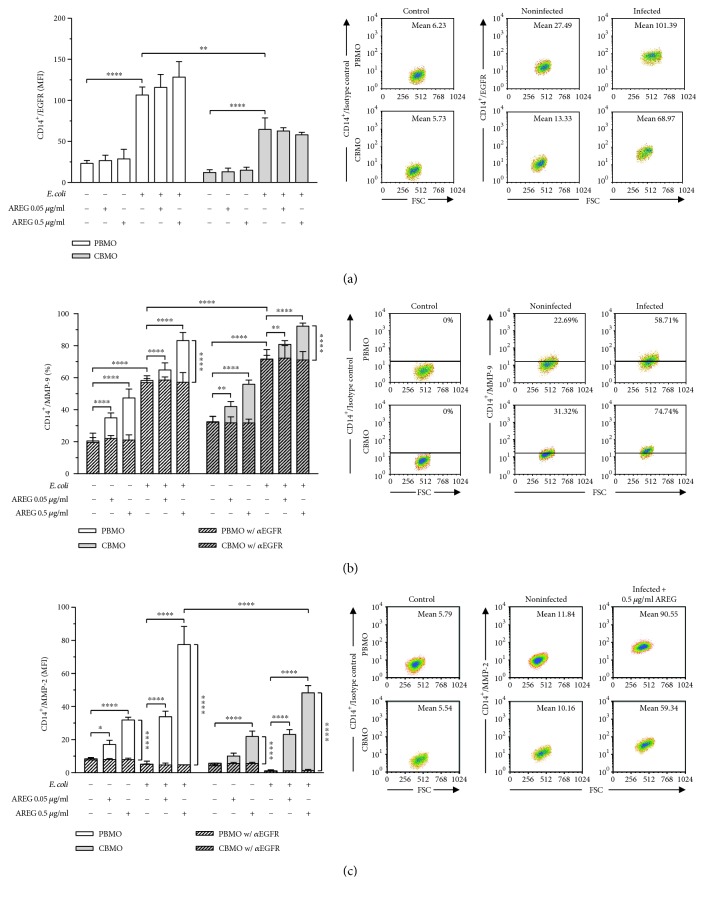
AREG induces protein expression of gelatinase enzymes through engagement with EGFR. PBMO and CBMO were infected as described in Figure 1. AREG stimulation and EGFR inhibitor treatment was started 1 h prior to infection and was maintained during cultivation. Representative dot plots show gating strategy and cutoff value. (a) EGFR surface expression in uninfected and *E. coli*-infected monocytes was analyzed in response to AREG stimulation by using flow cytometry. Increased EGFR levels were found in response to infection, while AREG stimulation remained without effect (*n* = 3). (b) Intracellular MMP-9 levels in uninfected and *E. coli*-infected monocytes were analyzed in response to AREG stimulation and neutralization of EGFR by using flow cytometry. MMP-9 was found increased in response to both infection and AREG stimulation, while the effect was abolished by concomitant EGFR inhibition (*n* = 5). (c) Intracellular MMP-2 levels in uninfected and *E. coli*-infected monocytes were analyzed in response to AREG stimulation and neutralization of EGFR by using flow cytometry. MMP-2 was increased in response to AREG stimulation, while infection remained without effect. The increase in MMP-2 levels was prevented by neutralization of EGFR (*n* = 5). Data are shown as means + SD. Statistical significance was analyzed by using two-way ANOVA with Bonferroni's multiple comparisons test (^∗^ *p* < 0.05, ^∗∗^ *p* < 0.01, ^∗∗∗^ *p* < 0.005, and ^∗∗∗∗^ *p* < 0.001).

Effector function of AREG is dependent on interaction with EGFR, leading us to comparatively analyze EGFR surface presentation in PBMO and CBMO. In both cell types, surface expression of EGFR was found significantly increased in response to *E. coli* infection. Furthermore, EGFR expression was overall higher in PBMO. As levels of surface EGFR remained unchanged in response to coincident AREG stimulation, we excluded the possibility that AREG affects intracellular signaling though upregulation of EGFR surface presentation (Figure 2(a)).

### 3.3. AREG Induces Protein Expression of MMP-9 and MMP-2

Since it has been shown that AREG regulates apoptosis of hepatocytes via memFasL shedding [30], we theorized that AREG might also affect PICD via regulation of extrinsic apoptosis signaling. The FasL/FasR system is one of the key apoptotic pathways, and evidence suggests that cytotoxic activity will only be exerted in case FasL is present in its membrane-bound form (memFasL) [40]. As matrix metalloproteases (MMPs) are known to mediate the conversion of memFasL into its inactive, soluble form (sFasL) [31, 32] and are coincidently regulated through EGFR/MAPK signaling, we next sought to analyze the two gelatinases MMP-2 and MMP-9 (Figures 2(b) and 2(c)). In both PBMO and CBMO, *E. coli* infection increased the portion of MMP-9-expressing cells but did not have an effect on MMP-2 protein expression. Stimulation with AREG revealed a dose-dependent increase in MMP-9 and -2 protein expression in both control and infected cells, indicating that AREG induces gelatinase protein expression. Notably, highest expression was observed after high-dose AREG stimulation and coincident infection. Neutralization of EGFR abolished the AREG-mediated increase in the proportion of MMP-9- and -2-expressing monocytes, supporting the idea of EGFR as a key component of this signaling mechanism.

### 3.4. AREG Promotes Shedding of memFasL in Infected Monocytes

**Figure 3:**
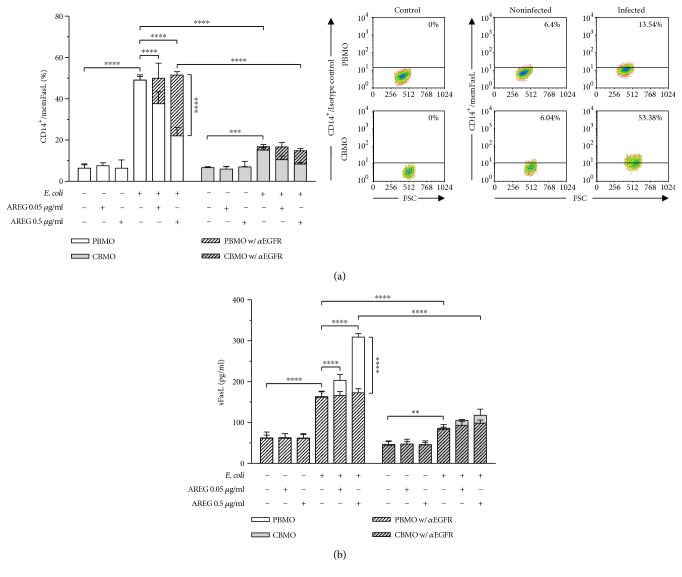
AREG mediates increased shedding of memFasL through engagement with EGFR. PBMO and CBMO were infected as described in Figure 1. AREG stimulation and EGFR inhibitor treatment was started 1 h prior to infection and was maintained during cultivation. (a) memFasL surface expression in uninfected and *E. coli*-infected monocytes was analyzed in response to AREG stimulation and neutralization of EGFR by using flow cytometry (*n* = 5). AREG stimulation significantly decreased FasL levels in infected PBMO but not CBMO. Neutralization of EGFR resulted in a complete rescue of infection-induced memFasL presentation. Representative dot plots show gating strategy and cutoff value. (b) sFasL levels in the supernatant of uninfected and *E. coli*-infected monocytes were quantified in response to AREG stimulation and neutralization of EGFR by using ELISA (*n* = 3). AREG significantly increased sFasL levels in infected PBMO but not CBMO, while neutralization of EGFR abolished the effect. Data are shown as means + SD. Statistical significance was analyzed using two-way ANOVA with Bonferroni's multiple comparisons test (^∗∗^ *p* < 0.01, ^∗∗∗^ *p* < 0.005, and ^∗∗∗∗^ *p* < 0.001).

**Figure 4:**
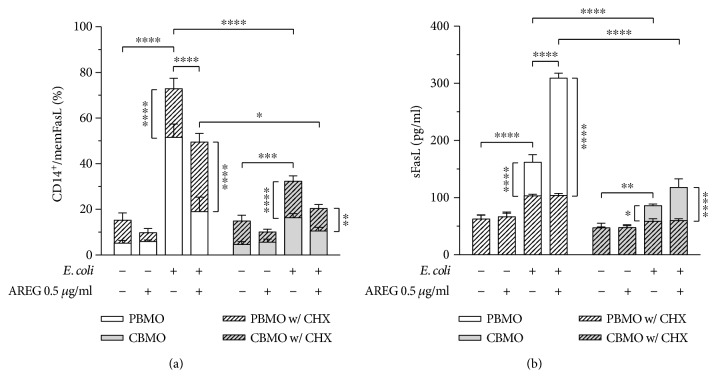
EGFR-dependent gelatinase activation mediates increased shedding of memFasL. PBMO and CBMO were infected as described in Figure 1. Stimulation with AREG and gelatinase inhibition by CHX treatment was started 1 h prior to infection and was maintained during cultivation. (a) memFasL surface expression in uninfected and *E. coli*-infected monocytes was analyzed in response to AREG stimulation and gelatinase inhibition by using flow cytometry (*n* = 4). Gelatinase inhibition prevented the decrease in memFasL presentation on monocytes triggered by AREG. (b) sFasL levels in the supernatant of uninfected and *E. coli*-infected monocytes were quantified in response to AREG stimulation and CHX treatment by using ELISA (*n* = 3). Gelatinase inhibition was found to prevent the increase in sFasL levels triggered by AREG. Data are shown as means + SD. Statistical significance was analyzed using two-way ANOVA with Bonferroni's multiple comparisons test (^∗^ *p* < 0.05, ^∗∗^ *p* < 0.01, and ^∗∗∗∗^ *p* < 0.001).

Our results demonstrate that AREG enhances gelatinase protein expression via engagement with EGFR. Given the involvement of both MMP-9 and -2 in FasL shedding, we next examined whether the AREG-mediated increase in both gelatinases indeed induces shedding of memFasL. As shown in Figure 3(a), the proportion of memFasL-displaying monocytes significantly increased in response to *E. coli* infection while the proportion was significantly higher in PBMO as compared to CBMO. Stimulation with AREG prevented the infection-induced increase in memFasL surface expression in PBMO by up to ~50%. When quantifying extracellular sFasL levels by ELISA, we found that infection resulted in significantly increased sFasL levels in both groups while the concentration was notably higher in PBMO as compared to CBMO (Figure 3(b)). In agreement with the decreased level of surface memFasL, AREG dose-dependently increased sFasL levels in infected PBMO but not CBMO. Neutralization of EGFR had no effect on memFasL or sFasL level under control conditions. As in infected cells, EGFR neutralization prevented the AREG-induced reduction of memFasL and the concurrent increase in sFasL, we speculated that AREG/EGFR engagement indeed causes gelatinase-induced memFasL cleavage. However, to find further evidence that FasL shedding is attributable to MMP-9 and -2, we used chlorhexidine (CHX) to neutralize the enzyme function [41] (Figure 4). In both infected and AREG-stimulated cells, gelatinase inhibition significantly increased the proportion of memFasL-displaying monocytes. Notably, we already detected a CHX-dependent increase in the percentage of memFasL-displaying cells under control conditions (*p* < 0.005). Corresponding to the increased memFasL surface expression, quantification revealed sFasL protein levels to be decreased in response to CHX treatment in both infected and AREG-stimulated monocytes. In order to exclude the possibility that either the neutralizing EGFR antibody or CHX affects the phagocytic capacity or shedding of pro-AREG, we examined monocyte apoptosis, pro-AREG expression, and soluble AREG levels but found all to be unaffected by either treatment (Supplementary Figure 1). Taken together, our findings indicate that FasL cleavage is indeed attributable to increased MMP-9 and MMP-2 protein levels induced by AREG/EGFR engagement.

### 3.5. AREG Stimulation Diminishes PICD of Monocytes

**Figure 5:**
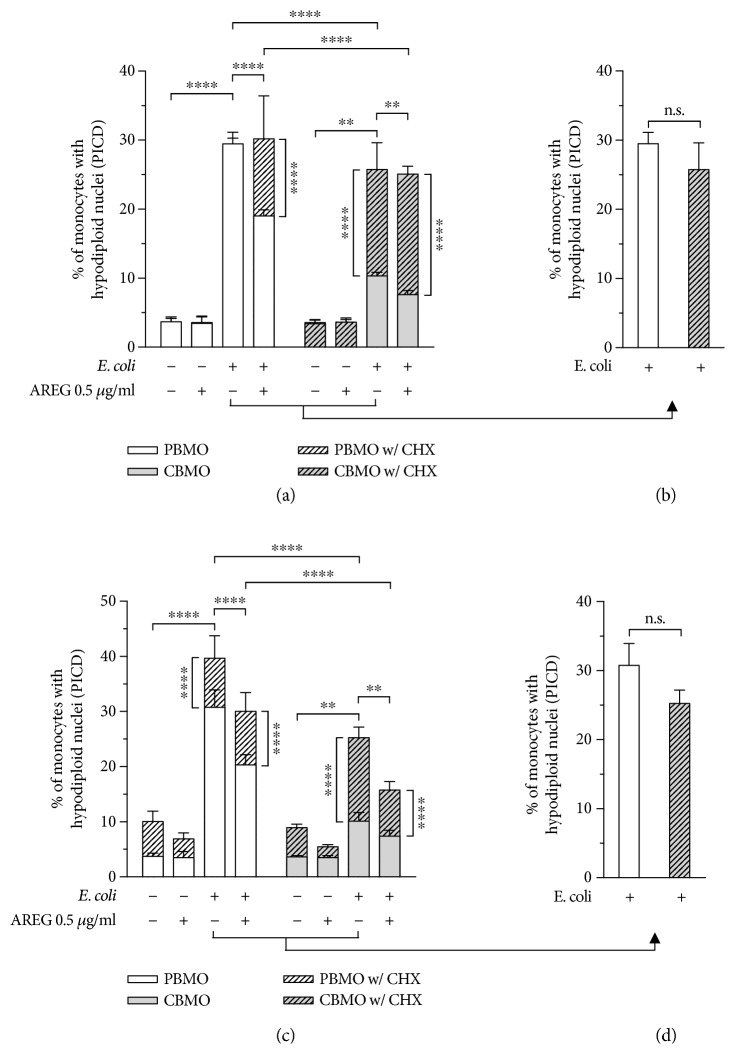
AREG/EGFR engagement reduces PICD in monocytes by elevating gelatinase-dependent memFasL shedding. PBMO and CBMO were infected as described in Figure 1. Neutralization of EGFR, stimulation with AREG, and gelatinase inhibition by CHX treatment was started 1 h prior to infection and was maintained during cultivation. Hypodiploid nuclei representing apoptosis induction in uninfected and *E. coli*-infected monocytes were analyzed in response to AREG stimulation by using flow cytometry. (a, b) AREG stimulation was found to selectively decrease PICD. Neutralization of EGFR resulted in a rescue of the suppressed PICD of neonatal monocytes (*n* = 4). (c, d) Gelatinase inhibition was found to abolish the difference in PICD between adult and neonatal monocytes (*n* = 4). Data are shown as means + SD of the percentage of CD14^+^ monocytes with hypodiploid nuclei as determined by PI staining. Data were analyzed using two-way ANOVA with Bonferroni's multiple comparisons test (ns: not significant; ^∗∗^ *p* < 0.01 and ^∗∗∗∗^ *p* < 0.001).

To demonstrate that AREG affects PICD in monocytes through engagement with EGFR and the involvement of gelatinase enzymes, we comparatively examined apoptosis of PBMO and CBMO (Figure 5). In both cell types, *E. coli* infection significantly increased the proportion of apoptotic cells, suggesting that these cells underwent PICD. Stimulation with AREG had no effect on control cells whereas it significantly decreased apoptosis in *E. coli*-infected PBMO and CBMO. These data argue for a selective involvement of AREG in PICD, rather than phagocytosis-independent apoptosis.

Neutralization of EGFR resulted in a complete rescue of the suppressed PICD in AREG-stimulated monocytes and, more strikingly, selectively increased the PICD of infected, unstimulated CBMO but not PBMO (Figures 5(a) and 5(b)). Direct comparison illustrates that inhibition of EGFR signaling abolished the difference in PICD between PBMO and CBMO. In both AREG-stimulated and unstimulated cells, inhibition of MMP-2 and -9 significantly increased PICD. Accordingly, gelatinase inhibition was also able to abolish the difference in PICD between PBMO and CBMO (Figures 5(c) and 5(d)). We additionally analyzed monocyte PICD upon infection with *S. agalactiae* and *C. albicans*, both well-known for being involved in severe neonatal infections (Supplementary Figure 2) [42]. Since we were able to confirm that stimulation with AREG reduces monocyte PICD, we concluded that the mechanism is not exclusive to monocyte infection with *E. coli* but generally applicable to microbial-induced PICD.

**Figure 6:**
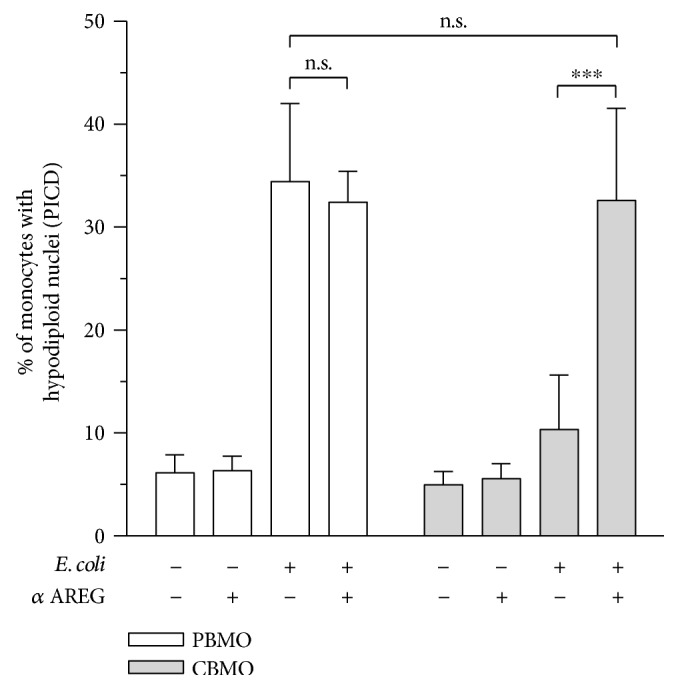
Loss of AREG offsets the difference in PICD of adult and neonatal monocytes. PBMO and CBMO were infected as described in Figure 1. Neutralization of AREG was started 1 h prior to infection and was maintained during cultivation. Hypodiploid nuclei representing apoptosis induction in uninfected and *E. coli*-infected monocytes were analyzed by using flow cytometry. Loss of AREG was found to selectively increase PICD of neonatal monocytes (*n* = 3). Data are shown as means + SD of the percentage of CD14^+^ monocytes with hypodiploid nuclei as determined by PI staining. Data were analyzed using one-way ANOVA with Bonferroni's multiple comparisons test (ns: not significant; ^∗∗^ *p* < 0.01 and ^∗∗∗∗^ *p* < 0.001).

The presented data strongly support the idea that AREG reduces extrinsic apoptosis in neonatal monocytes via gelatinase-induced FasL cleavage. However, to prove the specific effect of AREG and exclude the possibility of a parallel role of other EGFR ligands, we determined the consequences of the loss of AREG on monocyte PICD by using a neutralizing *α*AREG antibody. As shown in Figure 6, loss of AREG had no effect in control cells and did not affect PICD of PBMO. However, in line with our previous results, the proportion of CBMO that underwent PICD was found significantly increased upon AREG inhibition. Based on the finding that loss of AREG resulted in a complete rescue of the suppressed PICD of neonatal monocytes, we concluded that the previously demonstrated effects are attributable to AREG.

In summary, our data provide evidence that AREG reduces PICD of neonatal monocytes by enhancing gelatinase-mediated FasL cleavage. The insights obtained regarding the principles of phagocytosis-induced apoptosis signaling could be a helpful starting point for the development of new therapeutic strategies.

## 4. Discussion

An efficient and precise control of inflammation was shown to be particularly important for newborns due to the increased susceptibility of their developing organ system to inflammation-related damage [9, 43, 44]. Termination of immune effector cell viability is a critical step in the resolution of inflammation, suggesting PICD as an important event during inflammation. Since PICD of monocytes balances pro- and anti-inflammatory processes [45], the diminished PICD of neonatal monocytes may contribute to the prolongation of inflammation and the onset of neonatal inflammatory diseases, thereby highlighting the need to focus on the potential mechanism behind the reduced PICD in newborns. Although it has been shown through a large number of studies that AREG is expressed on various immune effector cells and is associated with the inflammatory response in several ways, there have been no studies addressing its role in monocyte PICD. To the best of our knowledge, this is the first study demonstrating distinct differences in pro-AREG presentation and shedding between adult and neonatal monocytes with 4-fold higher pro-AREG levels on monocytes of neonatal origin (Figure 1(b)). We further show that pro-AREG is cleaved from the cell surface in response to *E. coli* infection (Figure 1(c)), which is consistent with a number of studies demonstrating upregulation of AREG transcription as well as increased proteolytic cleavage of pro-AREG in response to various bacterial infections [20–23].

Effector function of AREG is dependent on engagement with EGFR [19]. We show that cell surface EGFR is increased in response to *E. coli* infection (Figure 2(a)), matching the results of another study that demonstrated upregulation of EGFR upon *Helicobacter pylori* infection [46]. It was already shown that TNF-*α* induces upregulation of EGFR [47], and in a previous study, we demonstrated that *E. coli* infection leads to TNF-*α* release, while PBMO displayed a distinctly increased TNF-*α* release compared to CBMO [48]. Therefore, we hypothesize that the increase in EGFR might be caused by *E. coli* infection and subsequent TNF-*α* release, thereby amplifying AREG function. However, as we did not specifically address the mechanism in the course of this study, future work will focus on clarifying the role of TNF-*α* in infection-mediated EGFR upregulation.

Since two major signaling routes of EGFR have been described to regulate protein expression of gelatinase enzymes [49], we started the examination of our hypothesis by analyzing MMP-9 and -2 (Figures 2(b) and 2(c)). Various studies demonstrated that activation of the EGFR-MAPK signaling pathway by shedding of diverse EGFR ligands leads to increased expression of MMP-2 and MMP-9 [50–53]. The results of our study demonstrate that AREG increases intracellular MMP-9 and -2 levels both under control conditions and upon infection, which is consistent with a previous study that showed AREG to upregulate MMP-9 in breast cancer cells [54]. It has been shown through a large number of studies that gelatinases are directly linked to apoptosis [55, 56] and more specifically, to FasL-mediated apoptosis [31, 32], leading us to analyze the impact of AREG on cellular memFasL and sFasL levels (Figures 3 and 4). Former work in hepatocytes revealed that AREG is capable of affecting apoptosis via memFasL shedding [30]. Matching these findings, we demonstrate that *E. coli* infection enhances both memFasL and sFasL levels. Strikingly, AREG stimulation diminished the levels of memFasl in infected monocytes, while leading to a concurrent increase in sFasL (Figures 3(a) and 3(b)). Inhibition of EGFR and MMP-2 and -9 prevented the increase in sFasL levels upon AREG stimulation, strongly supporting the idea that AREG decreases extrinsic apoptosis through gelatinase-mediated FasL shedding (Figures 3 and 4).

In addition to MMP-9 and -2, other matrix metalloproteases, such as MMP-7, were shown to mediate memFasL shedding [57]. Furthermore, we did not specifically address the question whether the increased MMP-9 and -2 levels actually reflect enzyme activity. Our data leave room for the possibility that other enzymes contribute to the cleavage of memFasL, and further analysis will be needed to identify other factors involved in this process and to prove whether the observed effects are attributable to increased enzyme activity. In line with the published literature, our data unveil 3-fold increased memFasL levels and 2-fold increased sFasL levels in response to infection in PBMO as compared to CBMO [45, 58, 59] which potentially contribute to the diminished PICD of neonatal monocytes (Figures 3(a) and 3(b)). Intriguingly, PICD reduction by AREG was only fully abolished when inhibiting EGFR but not MMP-9 and -2, suggesting that EGFR-dependent intrinsic apoptosis might also have relevance for AREG-dependent regulation of PICD. Strikingly, we show that loss of AREG rescues PICD in neonatal monocytes (Figure 6). However, even though this finding strongly supports the idea of a hitherto unrecognized functional involvement of AREG in monocyte PICD, the results do not necessarily exclude a parallel role of other EGFR ligands. Future studies are needed to prove that the observed effects are exclusively attributable to AREG.

Considering the present stage of our study, we can only speculate about the biological function of the higher AREG expression on neonatal monocytes. Given that newborns are characterized by rapid physical development, it is conceivable that the AREG/EGFR interaction contributes to the regulation of growth and organ functions. By influencing monocyte function and survival during infection, AREG potentially plays a role in shaping the response of other immune cells like T lymphocytes. At this point, we theorize that the temporary presence during the newborn period might identify AREG as endogenous immune regulator.

Notably, inhibition of MMP-2 and -9 was experimentally induced by CHX, which is a controversial inhibitor of gelatinase enzymes. Besides inhibition of MMP-9 and -2, CHX was also described to inhibit MMP-8 [41]. To address this, we used a final concentration of 0.002% CHX, thereby ensuring complete inhibition of MMP-2 und MMP-9 but maintaining MMP-8 function [41]. Nonetheless, a minor inhibitory effect on MMP-8 cannot be fully excluded. CHX was furthermore shown to have bactericidal properties [60–62]. However, although causing deformation in the bacterial cell wall and inhibiting proliferation, it does not affect the actual bacteria structure [60, 63]. As CHX does not have a cytotoxic effect on primary monocytes below a concentration of 30 *μ*g/ml [64] and remained without effect on phagocytosis, we are confident of the usability of CHX in the presented experimental setting.

Our study is based on an *in vitro* infection model applied to primary monocytes from peripheral or cord blood. Selection of monocytes was achieved by anti-CD14 coupled beads, leaving room for the possibility that the isolated cell fraction does not fully represent the actual *in vivo* situation. Although anti-CD14 antibodies were shown to particularly target monocytes, small reactivity on other cell types was also demonstrated [65]. Moreover, applicability of our data to the *in vivo* situation is potentially limited, given that working groups reported changes in monocyte antigen expression depending on the isolation method, assuming a slight differentiation after plating into culture plates [66]. Our experimental setting is based on *E. coli* as model organism, and even though we additionally showed that stimulation with AREG reduces monocyte PICD upon infection with *S. agalactiae* and *C. albicans* (Supplementary Figure 2), the role of AREG in these settings needs to be more thoroughly explored. Although *in vivo* experiments are clearly needed to clarify whether targeting AREG can prevent excessive inflammation and improve therapeutic outcomes in newborns, our data reveal a mechanistic role for AREG in the regulation of monocyte PICD.

## Data Availability

The data used to support the findings of this study are available from the corresponding author upon request.

## Abbreviations

*α*: Anti
ADAM-17: Disintegrin and metalloproteinase domain-containing protein 17
APC: Allophycocyanin
AREG: Amphiregulin
BSA: Bovine serum albumin
*C. albicans*: *Candida albicans*
CBMC: Cord blood mononuclear cells
CBMO: CD14-positive peripheral blood monocytes from cord blood
CD95L: CD95 ligand
CD95R: CD95 receptor
CHX: Chlorhexidine
DAPI: 4′,6-Diamidin-2-phenylindol
DMSO: Dimethyl sulfoxide
dsRed: Recombinant red fluorescent protein
*E. coli*: *Escherichia coli*
EGF: Epidermal growth factor
EGFR: Epidermal growth factor receptor
ELISA: Enzyme-linked immunosorbent assay
FasL: Fas ligand
FasR: Fas receptor
FBS: Fetal bovine serum
FITC: Fluorescein isothiocyanate
FSC: Forward scatter
IU: International unit
IgG: Immunoglobulin G
MAPK: Mitogen-activated protein kinase
MFI: Mean fluorescence index
MMP-2: Matrix metalloproteinase 2
MMP-9: Matrix metalloproteinase 9
MOI: Multiplicity of infection
PBMC: Peripheral blood mononuclear cells
PBMO: CD14-positive peripheral blood monocytes from adults
PBS: Phosphate-buffered saline
PBS-T: Phosphate-buffered saline Triton-X 100-supplemented
PI: Propidium iodide
PICD: Phagocytosis-induced cell death
RT: Room temperature
SD: Standard deviation
*S. agalactiae*: *Streptococcus agalactiae*
SSC: Sideward scatter
TNF-*α*: Tumor necrosis factor *α*.

## Supplementary Materials

Supplementary 1 Supplementary Figure 1: *α*EGFR and CHX treatment remains without effect on phagocytosis and pro-AREG shedding.

Supplementary 2 Supplementary Figure 2: AREG/EGFR engagement reduces PICD in monocytes by elevating gelatinase-dependent memFasL shedding.

Supplementary 3 Supplementary Figure 3: representative demonstration of the used strategy to quantify apoptotic monocytes.
