# Supplementary material for: Amphiregulin Regulates Phagocytosis-Induced Cell Death in Monocytes via EGFR and Matrix Metalloproteinases
Source: Mediators Inflamm. 2018 Nov 4;2018:4310419. doi: 10.1155/2018/4310419 (PMC6247478; doi:10.1155/2018/4310419)
Supplement: Supplementary 1 — Supplementary Figure 1: αEGFR and CHX treatment remains without effect on phagocytosis and pro-AREG shedding. [file 4310419.f1.pdf]

Supplementary Figure 1

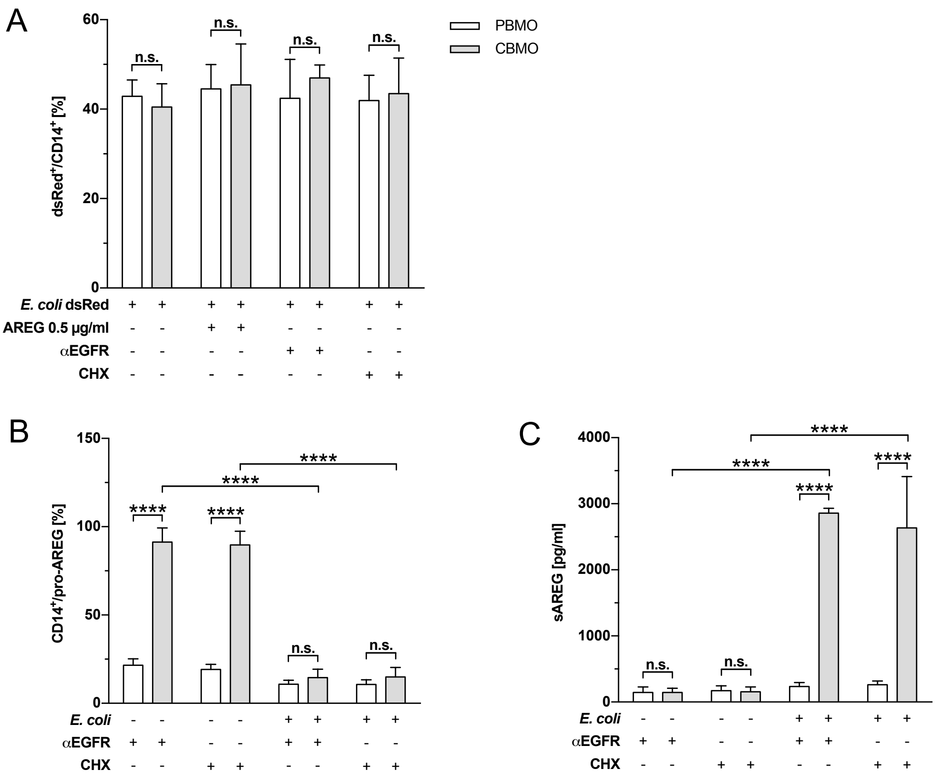

Suppl. Figure 1: αEGFR and CHX treatment remains without effect on phagocytosis and pro-AREG shedding. PBMO and CBMO were incubated with *E. coli* for 1 h, extracellular bacteria were removed and cells were cultivated for 24 h in total. Neutralization of EGFR, stimulation with AREG and gelatinase inhibition by CHX treatment was started 1 h prior to infection and was maintained during cultivation. A) Phagocytosis in uninfected and *E. coli*-infected monocytes was analyzed in response to AREG stimulation, neutralization of EGFR and gelatinase inhibition by using flow cytometry. Treatment remained without effect on phagocytic capacity (n=5). B+C) Pro-AREG surface expression and sAREG levels in the supernatant of uninfected and *E. coli*-infected monocytes was quantified by using flow cytometry respectively ELISA (n=5). Neutralization of EGFR and gelatinase inhibition remained without effect on pro-AREG shedding. Data were analyzed using two-way ANOVA with Bonferroni's multiple comparisons test (ns: not significant; ****p<0.001).
