# Supplementary material for: Amphiregulin Regulates Phagocytosis-Induced Cell Death in Monocytes via EGFR and Matrix Metalloproteinases
Source: Mediators Inflamm. 2018 Nov 4;2018:4310419. doi: 10.1155/2018/4310419 (PMC6247478; doi:10.1155/2018/4310419)
Supplement: Supplementary 2 — Supplementary Figure 2: AREG/EGFR engagement reduces PICD in monocytes by elevating gelatinase-dependent memFasL shedding. [file 4310419.f2.pdf]

Supplementary Figure 2

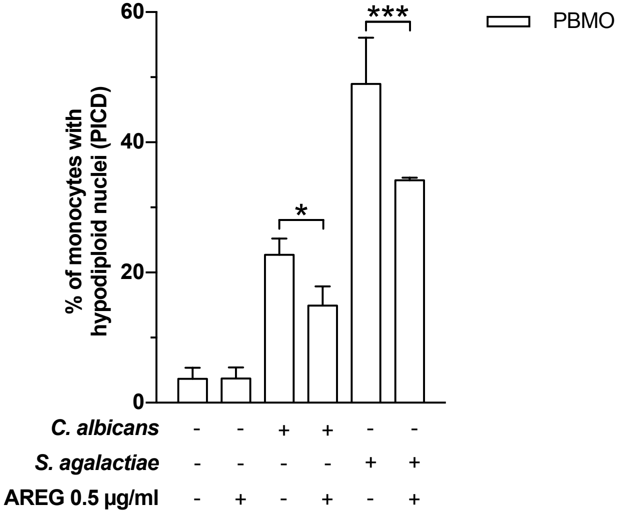

Suppl. Figure 2: AREG/EGFR engagement reduces PICD in monocytes by elevating gelatinase-dependent memFasL shedding. PBMO and CBMO were incubated with *S. agalactiae* and *C. albicans* for 1 h, extracellular microorganisms were removed and cells were cultivated for 24 h in total. Stimulation with AREG was started 1 h prior to infection and was maintained during cultivation. Hypodiploid nuclei representing apoptosis induction in uninfected and infected monocytes were analyzed by using flow cytometry. AREG stimulation was found to decrease PICD after infection with both *S. agalactiae* and *C. albicans*. Data are shown as means + SD of the percentage of CD14+ monocytes with hypodiploid nuclei as determined by PI staining. Data were analyzed using two-way ANOVA with Bonferroni's multiple comparisons test (ns: not significant; *$p<0.05$, ***$p<0.005$).
