# Supplementary material for: Amphiregulin Regulates Phagocytosis-Induced Cell Death in Monocytes via EGFR and Matrix Metalloproteinases
Source: Mediators Inflamm. 2018 Nov 4;2018:4310419. doi: 10.1155/2018/4310419 (PMC6247478; doi:10.1155/2018/4310419)
Supplement: Supplementary 3 — Supplementary Figure 3: representative demonstration of the used strategy to quantify apoptotic monocytes. [file 4310419.f3.pdf]

Supplementary Figure 3

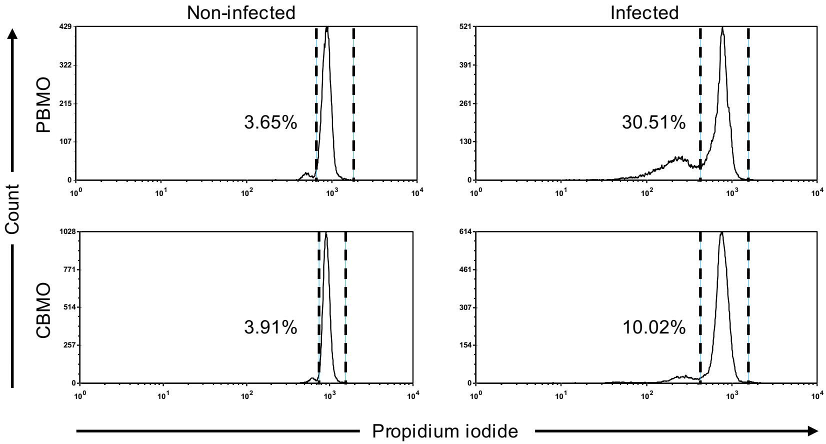

Suppl. Figure 3: Representative demonstration of the used strategy to quantify apoptotic monocytes. The illustration shows exemplary DNA fluorescence flow cytometric profiles of PI-stained monocytes after 1h *E. coli* infection and 24h cultivation. Panels on the left show DNA histograms of non-infected control cell and panels on the right show DNA histograms of infected cells without additional treatment.
